# The *Plasmodium falciparum* exported J domain proteins fine-tune human and malarial Hsp70s: pathological exploitation of proteostasis machinery

**DOI:** 10.3389/fmolb.2023.1216192

**Published:** 2023-06-30

**Authors:** Shaikha Y. Almaazmi, Rupinder P. Kaur, Harpreet Singh, Gregory L. Blatch

**Affiliations:** ^1^ Biomedical Research and Drug Discovery Research Group, Faculty of Health Sciences, Higher Colleges of Technology, Sharjah, United Arab Emirates; ^2^ The Department of Chemistry, Guru Nanak Dev University College Verka, Amritsar, Punjab, India; ^3^ Department of Bioinformatics, Hans Raj Mahila Maha Vidyalaya, Jalandhar, Punjab, India; ^4^ Biomedical Biotechnology Research Unit, Department of Biochemistry and Microbiology, Rhodes University, Grahamstown, South Africa

**Keywords:** heat shock proteins, J domain proteins, molecular chaperones and co-chaperones, *Plasmodium falciparum*, proteostasis, anti-malarial drug targets

## Abstract

Cellular proteostasis requires a network of molecular chaperones and co-chaperones, which facilitate the correct folding and assembly of other proteins, or the degradation of proteins misfolded beyond repair. The function of the major chaperones, heat shock protein 70 (Hsp70) and heat shock protein 90 (Hsp90), is regulated by a cohort of co-chaperone proteins. The J domain protein (JDP) family is one of the most diverse co-chaperone families, playing an important role in functionalizing the Hsp70 chaperone system to form a powerful protein quality control network. The intracellular malaria parasite, *Plasmodium falciparum*, has evolved the capacity to invade and reboot mature human erythrocytes, turning them into a vehicles of pathology. This process appears to involve the harnessing of both the human and parasite chaperone machineries. It is well known that malaria parasite-infected erythrocytes are highly enriched in functional human Hsp70 (HsHsp70) and Hsp90 (HsHsp90), while recent proteomics studies have provided evidence that human JDPs (HsJDPs) may also be enriched, but at lower levels. Interestingly, *P. falciparum* JDPs (PfJDPs) are the most prominent and diverse family of proteins exported into the infected erythrocyte cytosol. We hypothesize that the exported PfJPDs may be an evolutionary consequence of the need to boost chaperone power for specific protein folding pathways that enable both survival and pathogenesis of the malaria parasite. The evidence suggests that there is an intricate network of PfJDP interactions with the exported malarial Hsp70 (PfHsp70-x) and HsHsp70, which appear to be important for the trafficking of key malarial virulence factors, and the proteostasis of protein complexes of human and parasite proteins associated with pathology. This review will critically evaluate the current understanding of the role of exported PfJDPs in pathological exploitation of the proteostasis machinery by fine-tuning the chaperone properties of both human and malarial Hsp70s.

## 1 Introduction

The maintenance of cellular proteostasis depends on a network of molecular chaperones and co-chaperones which interact with proteins from “cradle to grave”, capturing nascent unfolded, partially folded or misfolded proteins and facilitating their fate whether it be folding or degradation (Edkins and Boshoff, 2021). They are therefore vital for ensuring that the structural integrity of the cellular protein machinery is maintained under normal physiological conditions, but especially under conditions of cell stress and diseased states (Wegele et al., 2004; Prodromou et al., 2023). The cellular functions of some of the major molecular chaperones, heat shock protein 70 (Hsp70) and heat shock protein 90 (Hsp90), are regulated by a cohort of co-chaperone proteins (e.g., Hsp70/Hsp90 organizing protein [HOP]; Odunuga et al., 2003; Bhattacharya and Picard, 2021; Schwarz et al., 2023; and J domain proteins [JDPs], also called heat shock protein 40 [Hsp40]; Hennessy et al., 2005; Kampinga and Craig, 2010; Zhang et al., 2023). Several different co-chaperone-regulated protein folding “pathways” have evolved, which are interconnected to form a well-organized chaperone network within the cell.

The JDP family is one of the most diverse co-chaperone families, with its membership far exceeding that of the Hsp70 family (Cyr and Ramos, 2023). Therefore, more than one JDP will service a particular Hsp70, and their primary role is to directly capture and handover client proteins, or bring them into close proximity, to partner Hsp70s (Cyr and Ramos, 2023). Consequently, the evolutionary radiation of the JDP family has functionalized the Hsp70 chaperone system to form a powerful protein quality control network contributing to cellular viability under normal, stressed and diseased states (Rosenzweig et al., 2019; Faust and Rosenzweig, 2020). In mechanistic terms, JDPs modulate the Hsp70 network to reduce proteotoxicity by preventing protein aggregation, solubilizing protein aggregates, promoting protein refolding, and directing recalcitrant misfolded proteins for degradation (Zhao et al., 2019; Sui et al., 2020; Chakraborty and Edkins, 2023).

**FIGURE 1:**
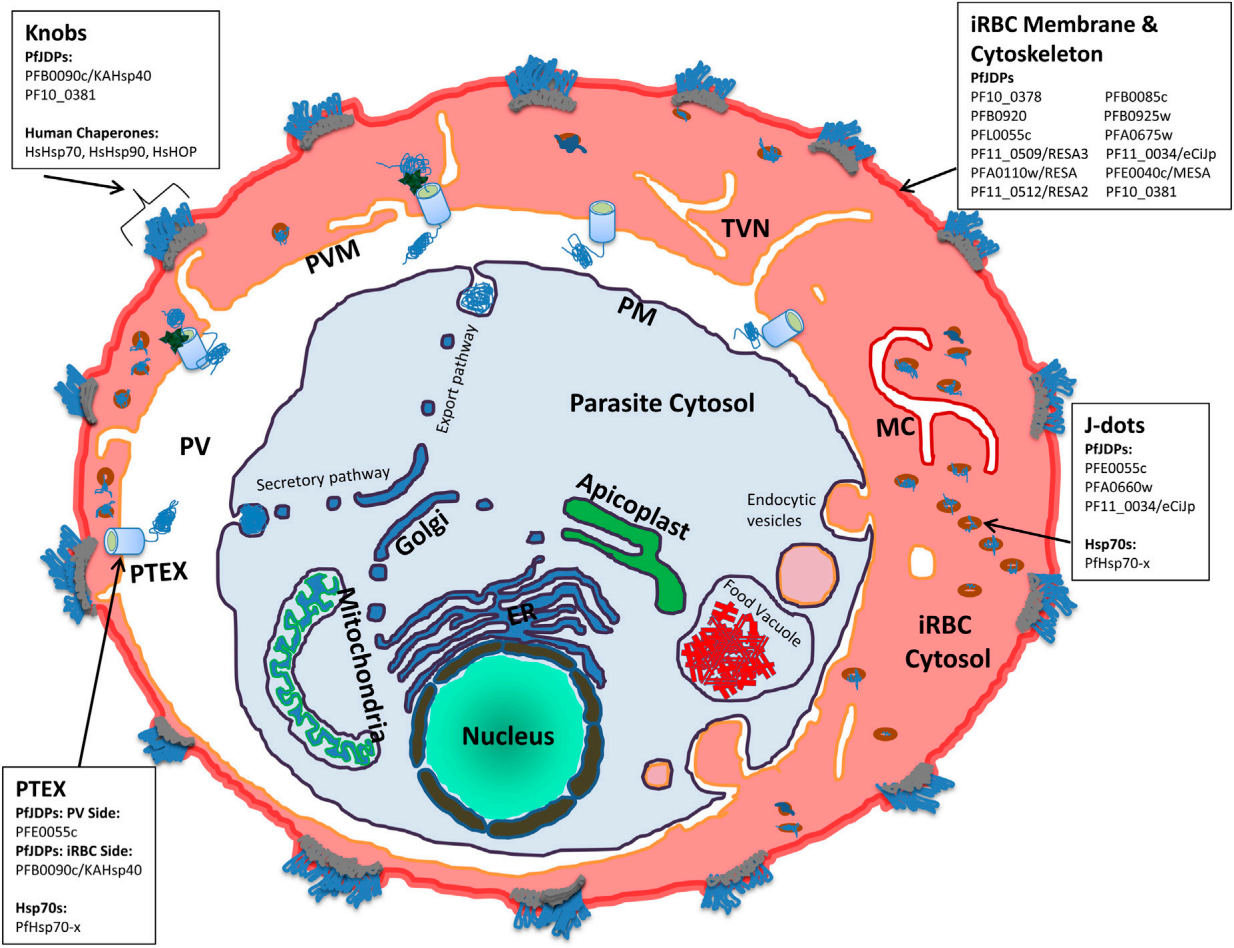
Schematic illustration of a *P. falciparum*-infected human erythrocyte showing the localization of human and exported malarial chaperones and co-chaperones. The compartments shown include the parasite (and associated organelles, and secretory and export pathways), parasitophorous vacuole (PV), the PV membrane (PVM), the *Plasmodium* translocon of exported proteins (PTEX), the infected red blood cell (IRBC) cytosol, membrane and cytoskeleton compartments, the tubovesicular network (TVN), Maurer’s Clefts (MC), knobs and J dot complexes. For the key protein complexes involved in the pathogenesis of malaria (PTEX, J dots, IRBC membrane and cytoskeleton, and knobs) the human (HsHOP, HsHsp70, and HsHsp90) and exported malarial chaperones (PfHsp70-x) and co-chaperones (PfJDPs) are indicated.

The life cycle of the malaria parasite, *Plasmodium falciparum*, involves a number of phases which traverse a human host and a mosquito vector. The parasite is able to escape exposure through a largely intracellular existence requiring highly regulated cycles of cellular invasion and egress (Dvorin and Goldberg, 2022). Malaria parasite-infected saliva is deposited into the human host when mosquitoes take a blood meal, releasing sporozoites which travel to the liver, where they develop into merozoites which are released into the blood stream where they invade erythrocytes. The clinical symptoms of malaria are associated with the erythrocytic phase, which involved multiple cycles of invasion, growth, multiplication, release and reinvasion of new erythrocytes. Some of the merozoites eventually develop into gametocytes, which can infect mosquitoes following another blood meal. Hence, a key phase in the pathology of malaria is the invasion of erythrocytes by the parasite (Figure 1). This intracellular parasite completely remodels the host cell by exporting around 450 parasite proteins, with heat shock proteins being a major component (∼5%) of the exportome (Jonsdottir et al., 2021). These heat shock proteins appear to play an important role in rebooting the protein folding capacity of the barren erythrocytic compartment, by establishing new co-chaperone-chaperone pathways involving both parasite and host protein machinery. The nature of these protein folding pathways have been explored in previous reviews (Cortés et al., 2020; Almaazmi et al., 2022; Blatch, 2022; Gabriela et al., 2022); however, there has been limited critique from a proteostasis and proteomics context. In this review, current proteostasis and proteomics perspectives are used to evaluate the role of exported PfJDPs in protein complexes involved in the pathology of malaria. The functionalization by PfJDPs of both human and malarial chaperones in these complexes is assessed, and the potential application of this knowledge in anti-malarial drug discovery is explored.

## 2 The structure of exported PfJDPs are similar but not identical to canonical JDPs

It is well known that the JDP-Hsp70 interaction is essential for stimulating the ATPase activity of Hsp70s (Liberek et al., 1991). Also, it is apparent from the above discussion that JDPs play a crucial role in precise control of the way Hsp70 interacts with a variety of substrates, thereby modulating Hsp70 function. The transient interaction of JDPs with ATP-bound Hsp70 is governed by their respective J domains (Craig and Marszalek, 2017; Hageman and Kampinga, 2009). The approximately 70-residue J domain is highly conserved among the JDPs, which otherwise show a variety of other functional/structural domains outside this region (Kampinga and Craig, 2010; Kampinga et al., 2019). This Hsp70-JDP J domains interaction, in coordination with substrate binding results in stimulation of the Hsp70 ATPase activity, leading to large scale conformational changes that finally stabilize substrate interaction (Clerico et al., 2009; Kampinga and Craig, 2010; Craig and Marszalek, 2017; Rosenzweig et al., 2019). The presence of a limited number of Hsp70s but a large number of JDPs in the cells hints that the diversity in Hsp70 functionality is largely driven by the JDPs (Kampinga and Craig, 2010). For example, the human genome encodes 50 JDPs as compared to only 17 Hsp70s, including 13 canonical Hsp70s and 4 non-canonical “Hsp70s” (the HSPH/Hsp110 family) exhibiting a longer linker region between the N-terminal nucleotide binding domain (NBD) and the C-terminal substrate binding domain (SBD) (Kampinga et al., 2009). The *P. falciparum* genome encodes at least 49 JDPs (PfJDPs; Botha et al., 2007; Njunge et al., 2013; Pesce and Blatch, 2014; Dutta et al., 2021a; Blatch, 2022), and at least six PfHsp70s, including four canonical Hsp70s (PfHsp70-1, PfHsp70-2, PfHsp70-3 and PfHsp70-x), and two non-canonical Hsp70s/Hsp110s (PfHsp70-y and PfHsp70-z) (Shonhai et al., 2007; Shonhai, 2021). Of the 49 PfJDPs, 18 are exported and tagged with an N-terminal PEXEL (*Plasmodium* export element) motif (Dutta et al., 2021a; Almaazmi et al., 2022), while of the six PfHsp70s, only one member has been found to be exported (PfHsp70-x), and interestingly does not have a PEXEL motif (Külzer et al., 2012; Grover et al., 2013).

**FIGURE 2:**
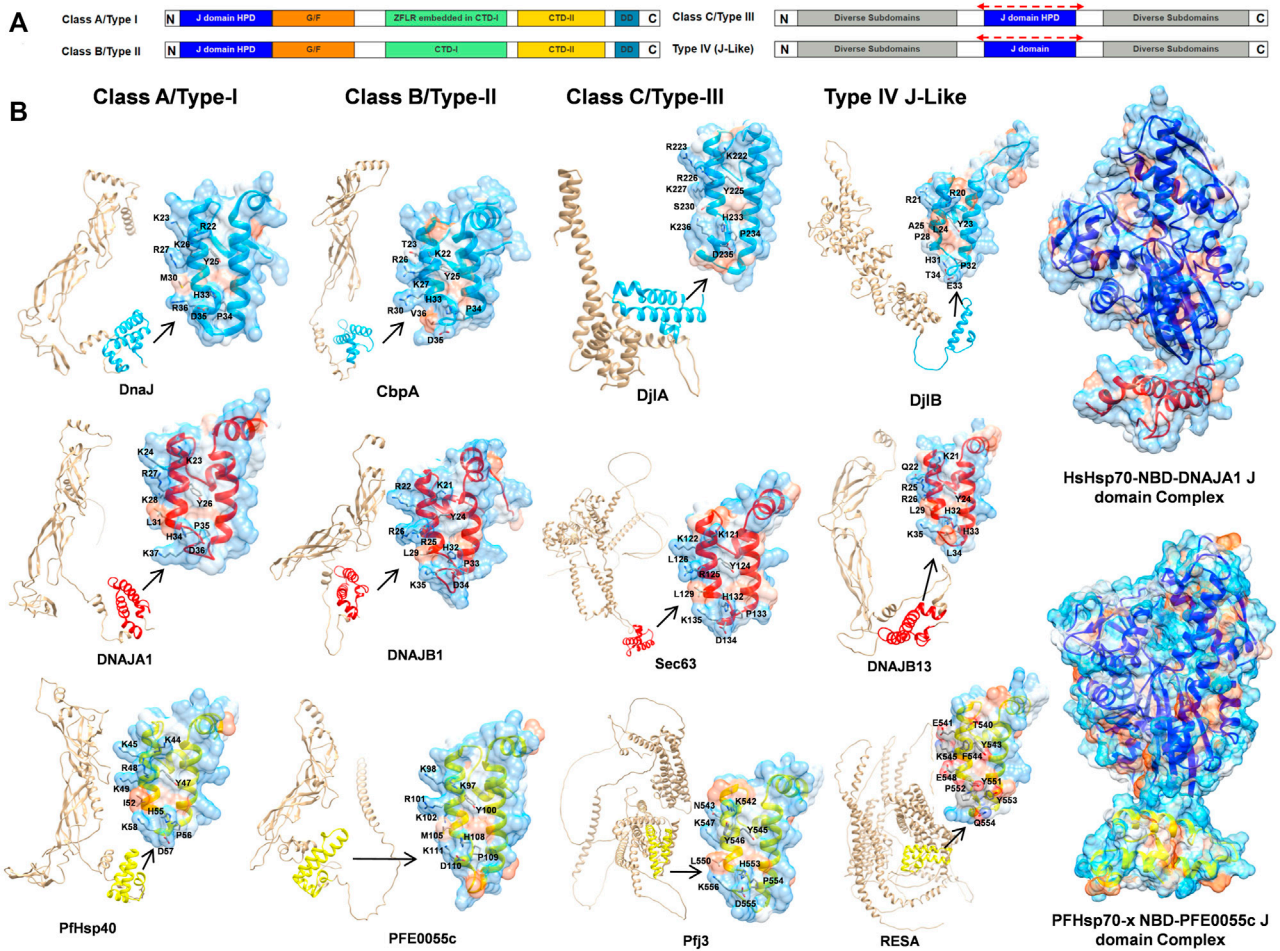
An overview of JDP classes/types and structural level conservation of the J domains. **(A)** The domain organization in various classes/types of JDPs, HPD: conserved histidine-proline-aspartic acid motif, G/F: glycine and phenylalanines rich region, CTDI: C terminal region 1, CTDII: C terminal region 2, ZFLR: zinc-finger-like region, DD: dimerization domain (Cheetham and Caplan, 1998; Botha et al., 2007; Kampinga et al., 2009; Njunge et al., 2013; Zhang et al., 2023). The red dotted lines on top of the J domain representation in class C/type-III and type-IV indicate that J domains may occur anywhere along the sequence. **(B)** Structural diversity of JDPs and relative occurrence of J domains among different classes. Well established representative members for each of the four classes/types were chosen for *E. coli*, human and *P. falciparum* (Supplementary Table S5) based on availability of high quality structural data/models and their role in proteostasis. Full-length predictions of JDPs by AlphaFold2 (Varadi et al., 2022) were obtained from the UNIPROT database (UniProt Consortium, 2015). Models for the J domains were obtained from these AlphaFold2 predicted models except for those with experimentally solved 3D structures viz., DnaJ (PDB ID: 5NRO; Kytik et al., 2015), CbpA (PDB ID: 2KQX; Sarraf et al., 2010), DNAJA1 (PDB ID: 2M6Y; Stark et al., 2014) and DNAJB1 (PDB ID: 1HDJ; Qian et al., 1996). The 3D structure of PFE0055c was earlier modeled by our group (Dutta et al., 2021b) using Modeller (Webb and Sali, 2016). The J domains have been colored blue, red and yellow for *E. coli*, human and *P. falciparum*, respectively. The zoomed in models of J domains depicts the hydrophobicity surface with 70% transparency to reveal the internal arrangement of secondary elements. The conserved residues important for J domain interaction with Hsp70 are shown as sticks colored by elemental type (Kityk et al., 2018). The HsHsp70-NBD DNAJA1 J domain (NBD: blue, J domain: red) and PfHsp70-x NBD-PFE0055c J domain (NBD: purple, J domain: yellow; Dutta et al., 2021b) complexes have been modelled using HADDOCK 2.4 (Van Zundert et al., 2016; Dutta et al., 2021b; Honorato et al., 2021). Depth-cued models, including the unprocessed versions of all the proteins have been rendered using UCSF Chimera 1.14 (Pettersen et al., 2004).

Historically, JDPs in general have been classified into three types based upon structural similarity to the *E. coli* DnaJ (EcDnaJ) (types I-III; Cheetham and Caplan, 1998). This initial classification was updated and adapted for human JDPs, resulting in three classes (class A, B and C; Kampinga et al., 2009; Zhang et al., 2023; Figure 2A). The members of class A enjoy full homology to EcDnaJ, consisting of the N-terminal J domain with the strictly conserved histidine-proline-aspartic acid (HPD) motif, a glycine/phenylalanine (G/F)-rich region, and a C-terminal region. The C terminal region further contains two β-sandwich subdomains called CTDI and CTDII, with the former harboring a zinc-finger-like region (ZFLR), and a dimerization domain. The class B consists of all the elements of class A, except the ZFLR. The members of class C are much more diverse sharing only the J domain with the EcDnaJ, with the J domain being one domain amongst a variety of other domains, with some of the members consisting only of the J domain (Zhang et al., 2023). The PfJDPs have been divided into four classes (types I-IV; Botha et al., 2007; Figure 2A), with types I-III being equivalent to human JDP classes A-C (Botha et al., 2007; Njunge et al., 2013). The type IVs have a J-like domain with a modified HPD motif (Botha et al., 2007). The types I-III together account for 36 PfJDPs while the remaining 13 of the PfJDPs are type IVs (Njunge et al., 2013; Dutta et al., 2021a). An overview of the full-length structural architecture and J domain features for all classes/types of JDPs is summarized in Figure 2B.

J domains typically consist of four helices (I-IV). The helices II and III connected by a loop form a finger like structure, an antiparallel coiled coil involving inter-chain hydrophobic interactions, while the other two helices I and IV protrude towards the end of the J domain providing stability to the overall J domain structure (Qian et al., 1996). The loop harbors the invariant HPD motif (Cheetham and Caplan, 1998; Cajo et al., 2006; Qiu et al., 2006) found in all the J domain classes except class IV (Botha et al., 2007), which is critical in catalytic stimulation of Hsp70 ATPase activity (Hennessy et al., 2005). Recently, Kityk et al. (2018) demonstrated that helix II residues 22 (R), 26 (K), and 27 (R) of the J domain of EcDnaJ play a crucial role in establishing interaction with the corresponding interface residues of the EcDnaK NBD. Notably, the HPD motif has been well conserved except in type IVs where residues of the HPD triad have been mutated (e.g., DjlB in *E. coli*, DNAJB13 in humans, and RESA in *P. falciparum*) (Figure 2B).

Hsp70s are also highly conserved, and consist of two large domains known as the N-terminal ATPase domain or NBD domain (40 kDa) and the C-terminal SBD, which are connected by a linker region (Bertelsen et al., 2009). The NBD allosterically controls the SBD which in turn binds to a short region of the substrate protein rich in hydrophobic residues (Rudiger et al., 1997; Mayer and Bukau, 2005). Binding of ATP to the NBD allows the domains to be docked such that the substrate binding site in the β subdomain of the SBD (SBDβ) is more accessible (Kityk et al., 2012; Qi et al., 2013). ATP hydrolysis leads to conformational changes allowing the α subdomain of the SBD (SBDα) to close the SBDβ site, thereby trapping the substrate (Mapa et al., 2010; Marcinowski et al., 2011; Banerjee et al., 2016). The interactions in this inter-domain interface play a key role in governing these conformational changes, specifically those at the base of the NBD with the linker and SBDβ (NBD/SBDβ, linker) (Vogel et al., 2006a; Zhuravleva et al., 2012; Kityk et al., 2015). The interactions of the NBD, SBDβ and linker with the J domain interface are critical for stimulation of ATPase activity of Hsp70s ( Jiang et al., 2007; Kityk et al., 2012; Qi et al., 2013; General et al., 2014).

In bacterial systems, the HPD motif of the J domain protrudes towards the linker region connecting the NBD and SBD, and is essential for stimulation of the Hsp70 ATPase activity (Wall et al., 1994; Tsai and Douglas, 1996; Campbell et al., 1997; Kelley and Georgopoulos, 1997; Gassler et al., 1998; Suh et al., 1998; Laufen et al., 1999; Mayer et al., 1999; Vogel et al., 2006b; Kumar et al., 2011). Recent studies have revealed three important interfaces marking the EcDnaJ-EcDnaK interaction (Malinverni et al., 2017; Kityk et al., 2018): the 1) J domain HPD catalytic interface with the NDB, linker region and SBD; and two critical binding interfaces important for the correct positioning of the catalytic HPD interface; with 2) the first involving helix II of the J domain and the lobe II of the NBD (the molecular structure of the J domain of EcDnaJ illustrating the contact residues, as well as and the topologically equivalent contact residues on the J domain of human and malarial JDPs, are illustrated in Figure 2B); and 3) the second involving the HPD motif and helix III of the J domain and the SBDβ. However, it is important to note that to the EcDnaJ-EcDnaK structure (Kityk et al., 2018) was obtained by fusing the first 105 residues (J domain and G/F rich) of EcDnaJ to the NBD of EcDnaK with a short linker, and hence might not have been able to capture the full landscape of the EcDnaJ-EcDnaK interface. Of all of the positively charged JDP J domain helix II residues involved in binding to the Hsp70 NBD, K/R26 (EcDnaJ numbering) has been found to be a highly conserved and functionally important residue in the vast majority of JDP proteins of prokaryotic, mammalian and parasitic origin (Hennessy et al., 2000; Genevaux et al., 2002; Hennessy et al., 2005; Nicoll et al., 2007). Interestingly, while the EcDnaJ-EcDnaK system appears to involve conserved positively charged J domain helix II residues (R22, K26 and R27) binding to both conserved (E217) and non-conserved (E206 and D211) negatively charged NBD residues (Kityk et al., 2018), molecular modelling studies on human and malarial JDP-Hsp70 complexes indicated that only conserved positively charged J domain helix II residues (R22 and K26; EcDnaJ numbering) and negatively charged residues of the Hsp70 NDB (D208, E209, and E217; EcDnaK numbering) were involved (Hatherley et al., 2005; Dutta et al., 2021b). This indicates that there may be key differences in the JDP-Hsp70 interface of eukaryotic systems compared to prokaryotic systems. Indeed, molecular modelling studies of the interaction of the key exported type II PfJDP, PFE0055c, suggests that it can interact stably with both the exported PfHsp70-x and HsHsp70 (Hatherley et al., 2005; Dutta et al., 2021b; Figure 2B); hinting at the possibility of being capable of functionalizing both the parasite and human Hsp70 systems. Furthermore, the exported type III and IV PfJDPs (e.g., Pfj3 and RESA, respectively; Figure 2B) have J domains where the helix II exhibits greater negative surface potential, particular the type IVs which also have a corrupt HPD motif. Overall, these data suggest that PfJDPs, especially the exported PfJDPs, are similar but not identical to the well-characterized bacterial JDPs, with unique features that suggest that they may fine-tune Hsp70s differently to canonical systems; however, experimental structure-function validation studies are required.

## 3 Human molecular chaperones are enriched in the infected erythrocyte cytosol

While the maturation of the human erythrocyte results in the degradation of many cytosolic proteins which remain as remnant peptides, a number of proteins remain folded and functional, including key molecular chaperones. Hence it is not surprising that the *P. falciparum*-infected erythrocyte cytosol has long been established to have significant levels of human Hsp70 (HsHsp70), Hsp90 (HsHsp90) and HOP (HsHOP) (Banumathy et al., 2002). In uninfected erythrocytes, HsHsp70 and HsHsp90 are largely cytosolic and soluble, whereas in infected cells a significant proportion of these chaperones are insoluble and associated with membranes and knobs (surface structures which present the cytoadherence receptor, *P. falciparum* erythrocyte membrane protein-1 (PfEMP1), a major virulence factor; Figure 1) (Banumathy et al., 2002). Furthermore, the membrane-associated HsHsp70 and HsHsp90 co-fractionated with the knob-associated protein, *P. falciparum* histidine rich protein 1 (PfHRP1) (Banumathy et al., 2002). Hence HsHsp70 is proposed to be involved in the formation of parasite complexes with erythrocyte cytoskeletal membrane proteins which are required for the loading of PfEMP1 on knobs (Banumathy et al., 2002). Indeed, detailed proteomics analyses of knobs have shown that it is a large structural complex of both human and malarial proteins, including human Hsp70 (HSPA8/Hsc70) (Alampalli et al., 2018; Figure 1). A number of proteomics studies of soluble and membrane-associated fractions of the cytosol of both uninfected and infected erythrocytes have identified human molecular chaperones (HsHOP, human JDPs [HsJDPs], HsHsp70 and HsHsp90) (Pasini et al., 2006; van Gestel et al., 2010; Bautista et al., 2014). Recent proteomics studies have provided semi-quantitative evidence that a number of HsHsp70 isoforms are enriched in the cytosolic soluble fraction of infected erythrocytes: HSPA1A/HSPA1B (HSP70-1; HSP72; HSPA1/HSP70-2); HSPA2 (Heat-shock 70kD protein-2); HSPA6/HSPA7 (Heat shock 70kD protein 6, HSP70B′/Heat shock 70kD protein 7); HSPA8 (HSC70; HSC71; HSP71; HSP73); and HSPH2 (HSPA4; APG-2; HSP110) (Supplementary Table S1; Siddiqui et al., 2022). As expected, HSPA1A/HSPA1B and HSPA8 were among the most enriched isoforms of HsHsp70. However, the non-canonical HsHsp70, HSPH2, was also highly enriched in the cytosolic soluble fraction of infected erythrocytes (Supplementary Table S1). This proteomics study also identified human Hsp90α (HsHsp90α) and HsHOP as highly enriched in the infected erythrocyte cytosolic soluble fraction, while HsHsp90β was an order of magnitude less enriched (Siddiqui et al., 2022). What about HsJDPs? While HsJDPs were not detected with as great an intensity as HsHOP, HsHsp70 and HsHsp90, members of all of the different HsJDP classes were identified as significantly enriched: DNAJA2 (DNJ3/mDJ3/Dnaj3/HIRIP4); DNAJA4 (HSJ4/Dj4); DNAJB1 (HSPF1/HSP40); DNAJB2 (HSJ1/HSPF3/Dnajb10/MDJ8); DNAJB4 (Hsc40); DNAJC9 (AU020082/RcDNAJ9); and DNAJC13 (Rme8/RME-8/Gm1124) (Supplementary Table S2; Siddiqui et al., 2022). While there is biochemical evidence that full length and functional HsHOP, HsHsp70 and HsHsp90 are present in the infected erythrocyte cytosol (Banumathy et al., 2002; Alampalli et al., 2018; Jonsdottir et al., 2021), there does not appear to be any such evidence for HsJDPs. The degree of enrichment of the HsJDPs detected by these various proteomics studies, suggests that they are functionally active; but this needs to be experimentally validated. Molecular chaperones and co-chaperones are one of the major families of exported proteins of the malaria parasite, with the malarial JDPs being the most prominent family of chaperones exported into the infected erythrocyte cytosol (which are reflected in the infected erythrocyte proteomics data; Supplementary Tables S3, S4; Siddiqui et al., 2022). This may seem surprising given the plethora of host chaperones potentially present in the infected erythrocyte. However, the exported JPDs may be an evolutionary consequence of the need to boost the proteostasis machinery, especially specific protein folding and complex assembly pathways that enable both survival and pathogenesis of the malaria parasite.

## 4 The exported PfJDP-Hsp70 chaperone network

As indicated in a previous section, the PfJDP family is highly expanded, with just under half predicted to be exported into the parasite-infected erythrocytes (Dutta et al., 2021a; Almaazmi et al., 2022; Blatch, 2022). A number of parasite-resident PfJDPs (PFL0565w/PF3D7_1211400/Pfj4; PF14_0359/PF3D7_1437900/PfHsp40; and PFB0595w/PF3D7_0213100) have been biochemically characterized and shown to functionally interact with the cytosolic and nuclear localized, highly abundant, and essential parasite-resident PfHsp70-1 (Pesce et al., 2008; Botha et al., 2011; Njunge et al., 2015). There is growing evidence that a number of the exported PfJDPs are networking with both the exported PfHsp70-x and HsHsp70 (Almaazmi et al., 2022; Blatch, 2022). Of the exported PfJDPs, there are three type IIs and four type IIIs, with the majority (11) being type IVs (Dutta et al., 2021a). Two of the exported type II PfJDPs (PFA0660w/PF3D7_0113700; and PFE0055c/PF3D7_0501100) have been shown to be specific co-chaperones of PfHsp70-x (Daniyan et al., 2016; Dutta et al., 2021b). PFE0055c, PFA0660w and PfHsp70-x have been shown to associate in highly mobile complexes (called “J dots”; Figure 1; Külzer et al., 2010; Külzer et al., 2012; Petersen et al., 2016). PfHsp70-x and J dot complexes have been shown to associate with the major virulence factor, PfEMP1, and hence they have been proposed to assist in the transport of PfEMP1 through the infected erythrocyte cytosol (Külzer et al., 2012; Behl et al., 2019). Furthermore, proteomics analysis of PfHsp70-x and PFE0055c complexes in the infected erythrocyte cytosol, identified two further J dot proteins, PfPHIST-0801 and PfGEXP18 (Zhang et al., 2017). PfPHIST_0801 is a poly-helical interspersed sub-telomeric sub-family c (PHISTc) member (PF3D7_0801000). PHIST proteins have been found associated with the erythrocyte membrane, knobs and the erythrocyte cytoskeleton, and it has been proposed that PHIST proteins may play a role in the transport and/or presentation of PfEMP1 on the surface of infected erythrocytes (Oberli et al., 2014; Oberli et al., 2016). These findings are consistent with a model in which the key J dot-associated PfJDPs, PFE0055c and PFA0660w, and PHIST collaborate with PfHsp70-x in the chaperoning and trafficking of PfEMP1 through the infected erythrocyte cytosol for insertion at the surface of knobs. PFE0055c is also present in the parasitophorous vacuole (PV; Figure 1), where it is associated with PfHsp70-x in high molecular weight complexes within this compartment (Zhang et al., 2017). Since PfHsp70-x has been found to interact with the *Plasmodium* translocon of exported proteins (PTEX) which resides in the PV membrane (PVM) (Elsworth et al., 2016; Zhang et al., 2017; Figure 1), it is tempting to speculate that PFE0055c serves as a co-chaperones of PfHsp70-x in the chaperoning of malarial proteins for delivery to PTEX for transport across the PVM into the erythrocyte cytosol. Interestingly, the third type II exported PfJDP, PFB0090c (PF3D7_0201800/KAHsp40), which is not associated with J dots, has also been shown to associate with the PTEX, and is proposed to be involved in the assembly of knob protein complexes (Acharya et al., 2012). Furthermore, deletion of the PFB0090c-encoding gene resulted in reduced cytoadherence (Maier et al., 2008). Since knobs contain HsHsp70 complexes with other human chaperones, it is not implausible that PFB0090c interacts either directly or indirectly with HsHsp70. Indeed, evidence is emerging that both PFE0055c (Zhang et al., 2017) and PFA0660w (Diehl et al., 2021) may also interact with HsHsp70.

While the type IV PfJDPs exhibit the greatest structural diversity and almost all are exported proteins (Botha et al., 2007; Njunge et al., 2013; Pesce and Blatch, 2014; Dutta et al., 2021a), little is known about the specific functions of these proteins (Almaazmi et al., 2022; Blatch, 2022). Since these PfJDPs have a corrupted HPD motif in their J domains, it is debatable whether or not they associate with Hsp70s. Indeed, helix II of the type IV PfJDP J domains also have reduced positively charged electrostatic potential compared to type I-III JDPs (Almaazmi et al., 2022; Figure 2B). Hence they appear to lack the two key components of the J domain required for binding and catalytic stimulation of Hsp70s. There is evidence that a number of the exported type IV PfJDPs are essential (e.g., PFB0085c/PF3D7_0201700 and PF14_0013/PF3D7_1401100; Zhang et al., 2018), required for survival under febrile conditions (e.g., PFA0110w/PF3D7_0102200/RESA; Silva et al., 2005; Diez-Silva et al., 2012), or involved in the formation of pathogenesis-related structures such as knobs (e.g., PF10_0381/PF3D7_1039100; Maier et al., 2008; Figure 1). Recently, an exported type IV PfJDP, called eCiJp (PF11_0034/PF3D7_1102200; a paralogue of PF10_0381), was found to be localized to J dots, associate with the erythrocyte cytoskeleton, and to potentially interact with HsHsp70 (HSPA1A) (Sahu et al., 2022). Therefore, overall the evidence suggests that there is an intricate network of known and still-to-be elucidated PfJDP interactions with PfHsp70-x and HsHsp70, within the parasite-infected erythrocyte cytosol, which are important for the trafficking of key malarial virulence factors. Furthermore, we propose that the interaction of exported PfJDPs with PfHsp70-x is critical to the trafficking, folding and functioning of malarial virulence factors (e.g., PfEMP1), while their interaction with HsHsp70 enables the formation and maintenance of protein complexes that support the pathology of malaria (e.g., cytoskeletal, membrane and knob complexes). Hence this network of exported PfJDP-Hsp70 interactions ensure proteostasis of the protein machinery needed to drive the pathology of malaria. All of these exported PfJDPs and PfJDP-Hsp70 partnerships represent potential drug targets for the development of anti-malarial drugs (Daniyan and Blatch, 2017).

## 5 The exported PfJDP-PfHsp70 complex is a potential drug target

While numerous inhibitors of Hsp70 ATPase and chaperone activities have been identified, very few small molecule compounds have been identified that modulate the functional interaction of JDPs with Hsp70s (Table 1). Small molecule compounds have been identified that: 1) inhibited JDP-stimulated ATPase activity and not the basal ATPase activity of Hsp70 by binding to an allosteric binding site on the NBD of Hsp70 (e.g., phenylmethyl ester/pyrimidinone MAL 3-101, Fewell et al., 2004; the flavonoid, myricetin, Chang et al., 2011); 2) that modulated JDP-stimulated ATPase activity of Hsp70 by binding to the JDP-binding site on Hsp70 (e.g., phenylmethyl ester/pyrimdinones 115-7c [stimulated] and 116-9e [inhibited]; Wisén et al., 2010); 3) inhibited JDP-stimulated ATPase activity and not the basal ATPase activity of Hsp70 by binding to an allosteric site on the JDP (e.g., phenoxy-N-arylacetamides; Cassel et al., 2012); 4) inhibited the JDP-stimulated chaperone activity of Hsp70 by binding to the substrate binding domain of the JDP (e.g., the D-peptide, R11-10, sequence VLARYLVQHV; Bischofberger et al., 2003); 5) inhibited JDP-Hsp70 physical interaction by stabilizing dimerization of the JDP (quercetin; Xu et al., 2010); and 6) inhibited JDP-Hsp70 physical interaction by binding to the J domain of the JDP (e.g., chalcone C86, a pan inhibitor, Moses et al., 2018; naphthoquinone/plumbagin derivatives, PLIHZ and PLTFBH; Alalem et al., 2022).

**TABLE 1:** Small molecule modulators of JDPs and their JDP-Hsp70 binding characteristics.

Chemotype	Common names	PubChem CID	Structure	System(s) used for studies	Binding characteristics	References
Phenylmethyl ester/pyrimidinone	MAL3-101	5461698	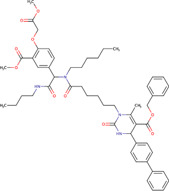	Yeast	Reduced the ability of J domain containing proteins to stimulate Hsp70 ATPase activity, and inhibited post-translational translocation of the protein	Fewell et al. (2004)
-	115-3b	5461601	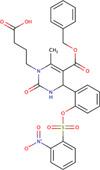	*Escherichia coli* and yeast	Mimics JDP by binding and activating JDP-Hsp70 complex	Wisén et al. (2010)
-	115-7c	5461551	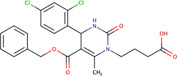			
-	116-9e	5461634	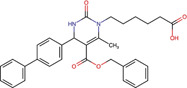	*Escherichia coli*	Interfere sterically with J domain interactions and inhibits the EcDnaJ-EcDnaK complex	Wisén et al. (2010)
Phenoxy-N-arylacetamide	-	1586557	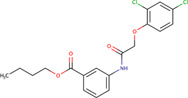	*Escherichia coli*	Inhibition of EcDnaJ-EcDnaK-mediated luciferase refolding through direct molecular interactions with DnaJ	Cassel et al. (2012)
-	KNK-437	24906297	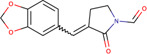	Human	A significant reduction in the level of DNAJA1 was observed, but no effect was observed on Hsp70 or Hsp90	Yang et al. (2020)
Chalcone	C86	3466699	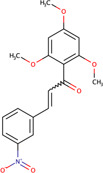	Human and mice	JDP pan-inhibitor, that interacts with DNAJA, DNAJB, and DNAJC proteins through their J domains	Moses et al. (2018)
Plumbagin-isonicotiyl hydrazide	PLIHZ	136264073	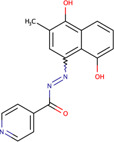	Human	Molecular docking studies indicate binding to the J domain of DNAJA1. Physical binding of PLIHZ to DNAJA1 in cells has been confirmed by a cellular thermal shift assay (CETSA)	Alalem et al. (2022)
Plumbagin-triflurobenzoic hydrazide	PLTFBH	-	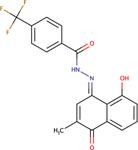			
Cabazitaxel	XRP-6258	9854073	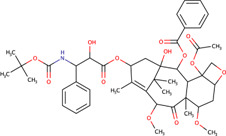	Human	Reduce JDP levels at very low doses	Rottach et al. (2019)
Tipifarnib	R-115777	159324	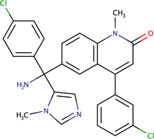	Human	Suppress levels of farnesylated HDJ-2 (homologue of DNAJ-2)	Wang et al. (2006)
Atorvastatin	-	60823	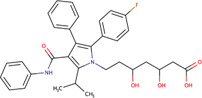	Human and mice	Inhibits the farnesylation of DNAJA1 in pancreatic cancer cells expressing wild-type or mutant p53 proteins	Xu et al. (2019)
Marbostat-100	M-100	118884610	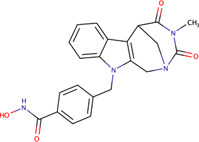	Human and mice	Inhibits HDAC6 by binding to DNAJA3 in hyperacetylated tubulin in B-cell lymphoma cells, which enhances the degradation of Myc	Winkler et al. (2022)
*Trans-*Chalcone	Cinnamoylbenzene	7189	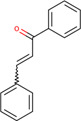	Human	Increases expression of JDP transcripts, thus increasing the protein’s level of expression	Silva et al. (2018)
*Trans-*Chalcone	T37	54523236	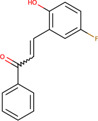			

There have been relatively few small molecule inhibitor studies on PfJDP-PfHsp70 interactions in general (Daniyan and Blatch, 2017; Barth et al., 2022), and even fewer studies that have identified specific small molecule inhibitors of the PfJDP-stimulated PfHsp70 ATPase activity or PfJDP-stimulated chaperone activity of PfHsp70s (Dutta et al., 2021b; Almaazmi et al., 2022). Small molecule compounds have been identified that inhibit the PfJDP-stimulated ATPase activity and not the basal ATPase activity of parasite-resident PfHsp70-1 (pyrimidinone DMT2264; Botha et al., 2011) and exported PfHsp70-x (malonganenone A; Cockburn et al., 2014). Furthermore, the chalcone C86, previously shown to bind to the J domain and serve as a pan-inhibitor of HsJDPs (Moses et al., 2018), was shown to inhibit the PFE0055c-stimulated ATPase activity of PfHsp70-x (Dutta et al., 2021b). This appears to be the only evidence of a small molecule inhibitor of J domain-based PfJDP functional interaction with PfHsp70. These studies are highly significant, since PFE0055c is essential to the survival of the malaria parasite, and because PFE0055c, PFA0660w and PfHsp70-x associate in J dots (Külzer et al., 2010; Külzer et al., 2012; Petersen et al., 2016) which are implicated in the trafficking of key virulence factors (Külzer et al., 2012; Behl et al., 2019), making them potential anti-malarial drug targets (Daniyan and Blatch, 2017; Dutta et al., 2021a; Almaazmi et al., 2022; Blatch, 2022).

**FIGURE 3:**
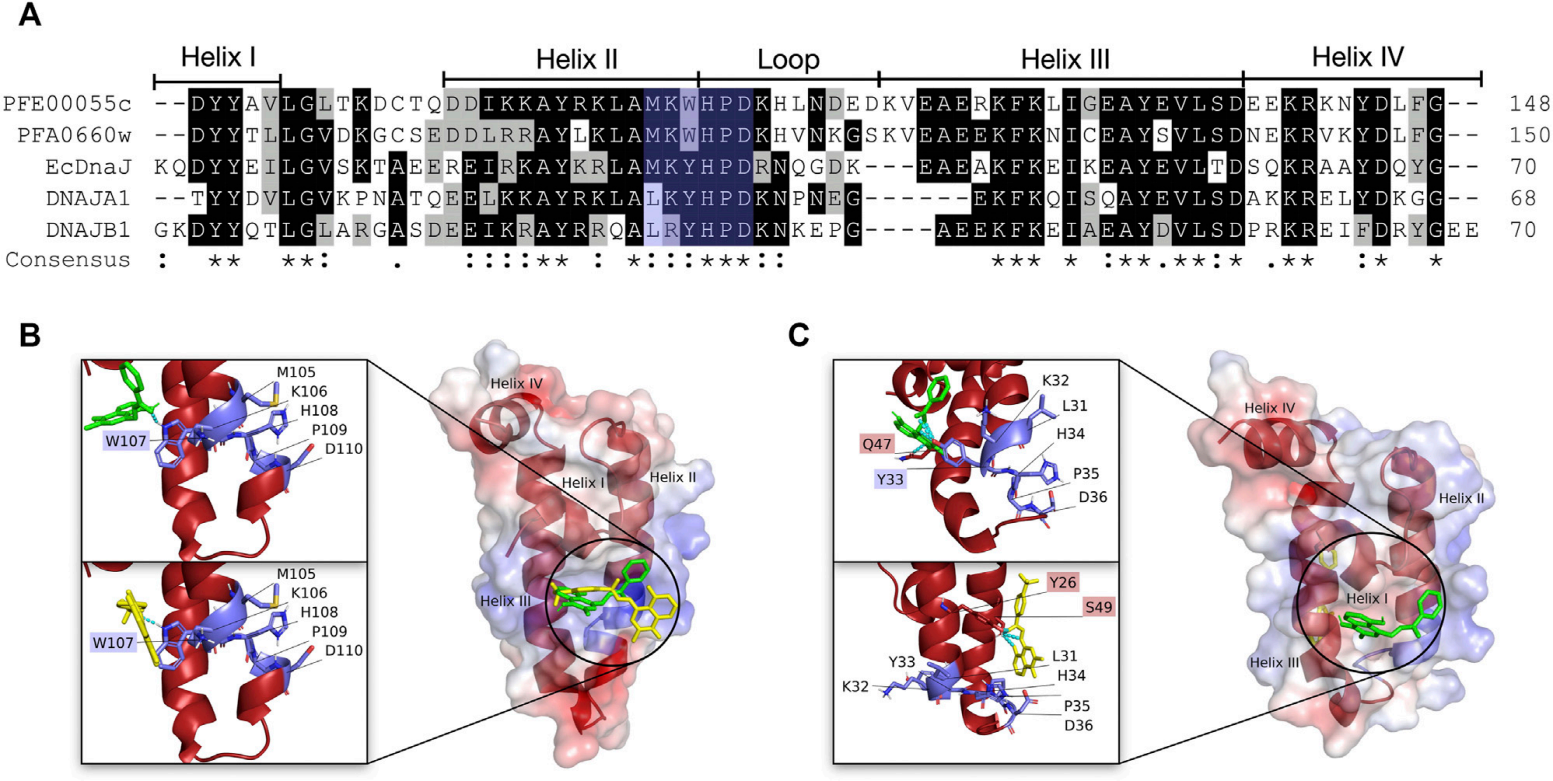
Multiple sequence alignment and three dimensional molecular docking highlighting the “MKWHPD” motif and docking of PLIHZ and PLTFBH on the J domain of the malarial PFE00055c and human DNAJA1. **(A)** Multiple sequence alignment of the J domains of the exported PfJDPs PFE00055c and PFA0660w, the human JDPs DNAJA1 and DNAJB1, and the *E. coli* JDP EcDnaJ. The proteins are defined by either their PlasmoDB accession number or common name in the first column. Colored in black are identical amino acids (in at least 50% of the aligned sequences), colored in light grey are similar amino acids (in at least 50% of the aligned sequences), and colored in white are the amino acids with no identity or similarity. The default categories for similar amino acids were applied to the multiple sequence alignment (ILV, FWY, KRH, DE, GAS, P, C, and M). The row titled “Consensus” are the common consensus symbols of the multiple sequence alignment: an “*” (asterisk) indicates positions which have a single, fully conserved residue; a “:” (colon) indicates conservation between groups of strongly similar properties; and a “.” (period) indicates conservation between groups of weakly similar properties. Highlighted with light blue shading is the “MKWHPD” motif. The alignment was created using Clustal Omega (Sievers and Higgins, 2018) and rendered with box shading using Multiple Align Show (Stothard, 2000). **(B)** The three dimensional structures of PLIHZ and PLTFBH docked onto the J domains of PFE00055c, and **(C)** DNAJA1. The protein structures in the middle of each figure show the docked PLIHZ as green sticks and PLTFBH as yellow sticks. The zoom-in on the left side of each protein structure show the “MKWHPD” motif as light blue sticks with the nitrogen, oxygen and sulfur highlighted with blue, red and orange colors, respectively. The cyan dotted lines represent hydrogen bonds between amino acid residues and the docked compounds, PLIHZ (green sticks) and PLTFBH (yellow sticks). Residue labels highlighted with light blue shading represent residues forming hydrogen bonds within the “MKWHPD” motif, while red shading represents residues forming hydrogen bonds outside the “MKWHPD” motif. The positive charge is shown in blue colored surface, negative charge shown in red colored surface and neutral potentials shown in white colored surface. The small molecule compounds were docked using Autodock Vina (https://vina.scripps.edu/; Trott and Olson, 2020), and the docking cluster was examined based on the lowest binding energy values (kcal/mol) and hydrogen interaction energies. The best pose from the cluster was selected out of three docking attempts, and based on appropriate pose orientation and the lowest binding energy. The surface electrostatic potential was calculated by APBS and graphically rendered using PyMol 2.5.2 (PyMOL Molecular Graphics System, Version 2.0 Schrödinger, LLC).

A molecular docking screen of the small molecule compounds known to directly modulate human and prokaryotic JDPs (Table 1), identified a number of compounds with potential preferential binding to the malarial system (PFE0055c J domain and PFE0055c-J domain-PfHsp70-x complex) over the human system (DNAJA1 J domain and DNAJA1-J domain-HsHsp70/HSPA1A complex). In particular, the plumbagin PLIHZ, and its derivative PLTFBH, exhibited selectivity toward the J domain of PFE0055c (Table 2). Interestingly, the docking poses indicated that while PLIHZ docked similarly on the PFE0055c and DNAJA1 J domains, its analogue PLTFBH docked similarly to PLIHZ on the J domain of PFE0055c, but had a completely different pose on the J domain of DNAJA1 (Figure 3). Both compounds were found to hydrogen bond with W107 on helix II of the J domain of PFE0055c, while only PLIHZ formed a hydrogen bond with the topologically equivalent Y33 of DNAJA1. Taken together these finding suggest that PLTFBH may preferentially bind to the J domain of PFE0005c over DNAJA1. The key W/Y residue was found by multiple sequence alignment to reside in an unexplored motif encompassing the conserved HPD motif (MKWHPD), which is present in all three of the exported type II PfJDPs (PFE0055c, PFA0660w and PFB0090c; Figure 3). Interestingly, the “W” is present in six of the 49 PfJDPs, exclusively type IIs and IIIs (PFE0055c, PFA0660w, PFB0090c, PFB0595w/PF3D7_0213100/PfSis1, PFL0565w/PF3D7_1211400/Pfj4 and PF11_0433/PF3D7_1142100), and 10 of the 50 HsJDPs, also exclusively type IIs and IIIs (DNAJB2, DNAJB3, DNAJB6, DNAJB7, DNAJB8, DNAJC3, DNAJC16, DNAJC21, DNAJC22, DNAJC28). The “MKWHPD” motif is a potential “signature” of the exported type II PfJDPs. Given that the EcDnaJ-EcDnaK structural analyses suggest that the HPD catalytic interface with EcDnaK involves the equivalent “MKYHPD” motif (specifically M30, H33, D35) (Kityk et al., 2018), PLIHZ and PLTFBH could interfere with JDP stimulation of Hsp70 ATPase activity. However, the functional importance of this “MKWHPD” motif remains to be experimentally elucidated.

**TABLE 2:** Docking binding energy values of JDP-Hsp70 modulators against modeled PFE00055c-PfHsp70-x (Dutta et al., 2021b) and DNAJA1-HsHsp70 complexes (modelled using HADDOCK 2.4), and the associated J domains alone using Autodock Vina. Binding energy values are represented as a “heat map” ranging from strong (red) to moderate (white) to weak (blue) binding values.

	Binding affinity of the top ranked conformation (kcal/mol)			
	PfHsp70-x-PFE00055c	HsHsp70-DNAJA1	PFE00055c J domain	DNAJA1 J domain
MAL3-101	−5.9	−6.3	−5.3	−5.5
115-3b	−5.9	−6.9	−6.2	−6.0
115-7c	−5.3	−5.9	−5.4	−4.6
116-9e	−6.7	−6.7	−6.1	−6.4
Phenoxy-N-arylacetamide	−6.2	−6.1	−6.3	−5.4
KNK-437	−5.8	−6.1	−6.1	−5.7
C86	−5.5	−5.5	−6.0	−5.3
PLIHZ	−6.2	−6.7	−6.9	−6.2
PLTFBH	−7.5	−7.8	−7.5	−6.8
XRP-6258	−7.1	−6.7	−6.6	−6.2
R-115777	−7	−6.7	−7.1	−6.5
Atorvastatin	−6.1	−6.3	−5.7	−5.5
M-100	−6.8	−7.6	−7.1	−6.3
Cinnamoylbenzene	−5.8	−5.9	−6.5	−6.0
T37	−6.0	−6.5	−6.5	−6.4

While these small molecule modulators of PfJDPs represent an exciting new horizon for anti-malarial drug discovery, further experimental studies are required. Using both *in vitro* and *in vivo* approaches, the affinity and specificity of these compounds (and chemo-type derivatives) for their targets needs to be validated, and their anti-malarial activity and cytotoxicity towards human cells tested. The elucidation of the crystal structure of PfJDP-PfHsp70 alone and in complex with these small molecule modulators and their derivatives would greatly enhance the identification of analogues with greater affinity and specificity. Ultimately, pre-clinical testing is imperative, to determine the effect of these modulators on the growth of malaria parasites using *in vitro* (*P. falciparum*-infected erythrocyte growth inhibition assays; and growth inhibition assays on clinical samples of infected erythrocytes) and *in vivo* assay (*P. falciparum* humanized mouse model) (Demarta-Gatsi et al., 2023).

## 6 Conclusion

There is now compelling evidence for an intricate network of exported PfJDP interactions with malarial and human Hsp70s, which plays a critical, and potentially essential, role in the trafficking, folding and structural presentation of key malaria virulence factors. The co-chaperone-chaperone pathways tailored to delivery of these virulence factors are starting to emerge, from their beginnings within the parasite, through the PV and coursing into the infected host cytosol to the cytoskeleton and membrane. J dots appear to be an important vehicle through which exported PfJDPs (especially PFE0055c and PFA0660w) collaborate with PfHsp70-x in the transport and chaperoning of PfEMP1 through the infected erythrocyte cytosol for insertion at the surface of knobs. But HsHsp70 may also be involved, given that both PFE0055c and PFA0660w have been shown to interact with this chaperone. The finding that PHIST family members associate with J dots, suggests that this complex could be involved in the trafficking of a number of malaria proteins destined for the cytoskeleton and membrane of infected erythrocytes. The role of PFE0055c goes beyond its involvement in J dots, as it is also present in the PV where it associates with PfHsp70-x in high molecular weight complexes potentially associated with the PTEX. Hence, PFE0055c may well be the major co-chaperone of PfHsp70-x in the chaperoning of malarial proteins for delivery to PTEX for transport across the PVM into the erythrocyte cytosol. However, many questions remain unanswered. What does the “chaperome” of the mature erythrocyte look like before and after malaria parasite invasion? What is the mechanism by which the exported PfJDPs harness the chaperone power of HsHsp70s, and are the type IV PfJDPs involved? What structural features of exported PfJDPs and PfJDP-Hsp70 partnerships could be exploited for the development of novel anti-malarial drugs? May these and the many other questions be answered in the near future.
