# Supplementary material for: The Plasmodium falciparum exported J domain proteins fine-tune human and malarial Hsp70s: pathological exploitation of proteostasis machinery
Source: Front Mol Biosci. 2023 Jun 30;10:1216192. doi: 10.3389/fmolb.2023.1216192 (PMC10349383; doi:10.3389/fmolb.2023.1216192)
Supplement: Supplementary file 1 [file Table1.DOCX]

Supplementary Material

The *Plasmodium falciparum* exported J domain proteins fine-tune human and malarial Hsp70s:

Pathological exploitation of proteostasis machinery

Shaikha Y. Almaazmi^1^, Rupinda Preet Kaur^2^, Harpreet Singh^3^, Gregory L. Blatch^1,4*^

*** Correspondence:** Gregory L. Blatch: g.blatch@ru.ac.za

# Supplementary Tables

**Supplementary Table S1:** Human Hsp70, Hsp90 and HOP detected by proteomic analyses of *P. falciparum-*infected erythrocytes (extracted from Siddiqui et al. 2022)

| **Formal Name**  **(Old Name)** | **Uniprot ID** | **Relative Protein Intensities** | | | |
| --- | --- | --- | --- | --- | --- |
|  |  | **Rings** | **Trophozoites** | **Schizonts** | **Supernatant** |
| HSPA1A/HSPA1B  (HSP70-1; HSP72; HSPA1/HSP70-2) | P0DMV9/P0DMV8 | 1342500000 | 10399000000 |  |  |
|  |  |  |  | 1884600000 | 18341000000 |
| HSPA2 (Heat-shock 70kD protein-2) | P54652 |  | 211960000 |  | 129250000 |
| HSPA6/HSPA7  (Heat shock 70kD protein 6, HSP70B′/Heat shock 70kD protein 7) | P17066/P48741 | 14483000 | 238150000 | 184990000 | 219470000 |
| HSPA8  (HSC70; HSC71; HSP71; HSP73) | P11142 | 4335500000 |  |  |  |
|  |  |  |  | 71764000000 |  |
|  |  |  | 11922000000 | 5633500000 | 64823000 |
|  |  |  |  |  | 37937000000 |
|  |  |  | 1.97E+11 |  |  |
| HSPH2 (HSPA4; APG-2; HSP110) | P34932 |  |  |  | 18436000000 |
| HSPC2 (Hsp90α) | P07900 |  | 7002800 |  | 10742000000 |
| HSPC2/HSPC3 (Hsp90α/Hsp90β) | P07900/P08238 | 18981000 |  |  |  |
| HSPC3 (Hsp90β) | P08238 |  | 402940000 |  | 464770000 |
| HOP (STI1; Stip1; p60) | P31948 |  |  | 20948000 |  |
|  |  |  |  |  | 31453000000 |
|  |  |  | 71123000 |  |  |

**Supplementary Table S2:** Human JDPs detected by proteomic analyses of *P. falciparum-*infected erythrocytes (extracted from Siddiqui et al. 2022)

| **Formal Name**  **(Old Name)** | **JDP Type** | **Uniprot IDs** | **Relative Protein Intensities** | | | |
| --- | --- | --- | --- | --- | --- | --- |
|  |  |  | **Rings** | **Trophozoites** | **Schizonts** | **Supernatant** |
| DNAJA2  (DNJ3/mDJ3/Dnaj3/HIRIP4) | I | O60884 |  |  |  | 15312000 |
| DNAJA4 (HSJ4/Dj4) | I | Q8WW22 |  | 281170000 | 23869000 |  |
|  |  |  |  |  |  | 1004700000 |
| DNAJB1  (HSPF1/HSP40) | II | P25685 | 10671000 |  |  |  |
|  |  |  |  |  |  | 2372600000 |
| DNAJB2  (HSJ1/HSPF3/Dnajb10/MDJ8) | II | P25686 | 2120300 |  |  |  |
|  |  |  |  | 325410000 | 57814000 | 1220700000 |
| DNAJB4  (Hsc40) | II | Q9UDY4 |  |  |  | 239650000 |
| DNAJB11  (Dj9/ABBP-2/Erdj3) | II | Q9UBS4 |  | 5975500 |  |  |
| DNAJC9  (AU020082/RcDNAJ9) | III | Q8WXX5 | 41175000 |  |  | 567700000 |
| DNAJC13  (Rme8/RME-8/Gm1124) | III | O75165 |  |  |  | 501790000 |
|  |  |  |  | 3616800 |  |  |

**Supplementary Table S3:** PfHOP, PfHsp70s and PfHsp90s detected by proteomic analyses of *P. falciparum-*infected erythrocytes (extracted from Siddiqui et al. 2022)

| **PlasmoDB ID Old** | **PlasmoDB ID New** | **Common Name** | **Relative Protein Intensities** | | | |
| --- | --- | --- | --- | --- | --- | --- |
|  |  |  | **Rings** | **Trophozoites** | **Schizonts** | **Supernatant** |
| PF14_0324 | PF3D7_1434300 | PfHOP | 106460000 | 40425000000 | 22224000000 | 2042500000 |
| PF08_0054 | PF3D7_0818900 | PfHsp70-1 | 5764000000 | 4.81E+11 | 5.91E+11 | 14719000000 |
| PFI0875w | PF3D7_0917900 | PfHsp70-2 | 12308000000 | 7.03E+11 | 6.70E+11 | 29239000000 |
| PF11_0351 | PF3D7_1134000 | PfHsp70-3 | 1232600000 | 2.46E+11 | 1.45E+11 | 659510000 |
| MAL7P1.228 | PF3D7_0831700 | PfHsp70-x | 117670000 | 68256000000 | 31613000000 | 34276000000 |
| MAL13P1.540 | PF3D7_1344200 | PfHsp70-y | 105640000 | 12171000000 | 28405000000 |  |
| PF07_0033 | PF3D7_0708800 | PfHsp70-z | 651860000 | 36862000000 | 60153000000 | 10416000000 |
| PF14_0417 | PF3D7_1443900 | PfHsp90_A |  | 13405000000 | 10258000000 |  |
| PF07_0029 | PF3D7_0708400 | PfHsp90 | 2393000000 | 3.02E+11 | 3.16E+11 | 68636000000 |
| PFL1070c | PF3D7_1222300 | PfGrp94 | 8285300000 | 4.33E+11 | 2.02E+11 | 56270000 |
| PF11_0188 | PF3D7_1118200 | PfTrap1 | 46882000 | 30096000000 | 25372000000 |  |

**Supplementary Table S4:** PfJDPs detected by proteomic analyses of *P. falciparum-*infected erythrocytes (extracted from Siddiqui et al. 2022)

| **PlasmoDB ID**  **Old** | **PlasmoDB ID**  **New** | **Common Name** | **Relative Protein Intensities** | | | | | |
| --- | --- | --- | --- | --- | --- | --- | --- | --- |
|  |  |  | **Rings** | **Trophozoites** | **Schizonts** | **Supernatant** | **JDP**  **Type** | **PEXEL** |
| PFE0040c | PF3D7_0500800 | MESA/  PfEMP2 | 20578000 | 1.48E+11 | 2.27E+11 | 51945000000 | IV | Yes |
| PF14_0013 | PF3D7_1401100 |  |  | 223250000 | 101020000 | 9131200000 | IV | Yes |
| PFL2550w | PF3D7_1253000 | PfGECO |  | 239950000 |  | 850250000 | IV | Yes |
| PF14_0359 | PF3D7_1437900 | PfHsp40 |  | 10466000000 | 22055000000 | 402440000 | I | No |
| PFA0110w | PF3D7_0102200 | RESA | 420440000 | 22611000000 | 5224800000 | 394440000 | IV | Yes |
| PFA0660w | PF3D7_0113700 |  |  |  |  | 131970000 | II | Yes |
| PFL0565w | PF3D7_1211400 | Pfj4 |  | 4483500000 | 7058700000 | 97875000 | II | No |
| PFB0595w | PF3D7_0213100 | PfSis1 | 20539000 | 6349800000 | 4620100000 | 88102000 | II | No |
| PFE0055c | PF3D7_0501100 |  | 87004000 | 774840000 | 2832600000 | 38861000 | II | Yes |
| PFB0925w/  PF08_0115 | PF3D7_0220400/PF3D7_0806500 |  |  |  |  | 35994000 | IV/III | Yes/No |
| PFB0085c | PF3D7_0201700 |  |  |  |  | 27514000 | IV | Yes |
| PF14_0700 | PF3D7_1473200 |  | 36971000 | 15213000000 | 11294000000 | 24067000 | III | No |
| PFB0090c | PF3D7_0201800 | KAHsp40 |  |  | 1166000000 | 22095000 | II | Yes |
| PFD0462w | PF3D7_0409400 | Pfj1 |  | 795280000 | 692660000 |  | I | No |
| PFF1415c | PF3D7_0629200 |  | 130480000 | 29953000000 | 16284000000 |  | II | No |
| PF11_0099 | PF3D7_1108700 | Pfj2 | 35278000 | 15787000000 | 14195000000 |  | II | No |
| PF14_0137 | PF3D7_1413900 |  |  | 124170000 | 877750000 |  | II | No |
| PF08_0032 | PF3D7_0823800 |  | 100340000 | 16873000000 | 12673000000 |  | III | No |
| PFE1170w | PF3D7_0523400 |  | 24581000 | 3293700000 | 1658600000 |  | III | No |
| PF08_0115 | PF3D7_0806500 |  |  | 617950000 | 584650000 |  | III | No |
| MAL9P1.204 | PF3D7_0831200 |  |  | 56697000 | 23663000 |  | III | No |
| PFI0855w | PF3D7_0917500 |  |  | 1443300000 | 1515900000 |  | III | No |
| PFI0935w | PF3D7_0919100 |  |  | 7954200000 | 13188000000 |  | III | No |
| PFI0985c | PF3D7_0920100 | PfJac1 |  | 79743000 | 275740000 |  | III | No |
| PF10_0057a | PF3D7_1005600 | PfJjj1 |  |  | 16875000 |  | III | No |
| PF11_0380 | PF3D7_1136800 |  |  | 1628500000 | 2147300000 |  | III | No |
| PFL0815w | PF3D7_1216900 | PfZuo1 |  | 4464300000 | 1678300000 |  | III | No |
| PF13_0036 | PF3D7_1307200 |  |  |  | 299270000 |  | III | No |
| MAL13P1.162 | PF3D7_1330300 |  |  | 572150000 | 215300000 |  | III | No |
| PF14_0111 | PF3D7_1411300 |  |  | 107720000 | 8365200 |  | III | No |
| PF10_0032 | PF3D7_1002800 |  |  | 281300000 |  |  | III | No |
| PF11_0273 | PF3D7_1126300 |  |  | 60355000 |  |  | III | No |
| PF14_0213 | PF3D7_1422300 |  |  | 96506000 |  |  | III | No |
| PF13_0102 | PF3D7_1318800 | PfSec63 | 593350000 | 14097000000 | 17999000000 |  | III | No |
| PF11_0433 | PF3D7_1142100 |  |  | 4480700000 | 2866200000 |  | III | No |
| PF07_0103 | PF3D7_0724400 | PfPam18/  Tim14 |  | 344530000 |  |  | III | No |
| PFF1010c | PF3D7_0620700 |  |  | 154980000 | 48817000 |  | IV | No |
| PF11_0443 | PF3D7_1143200 |  |  | 773270000 | 1322400000 |  | IV | No |
| PFB0920w | PF3D7_0220100 |  |  | 602890000 | 12358000 |  | III | Yes |
| PFL0055c | PF3D7_1201100 |  |  | 701680000 | 68224000 |  | III | Yes |
| PF11_0513 | PF3D7_1149600 |  |  | 193420000 |  |  | III | Yes |
| PF11_0509 | PF3D7_1149200 | RESA3 | 94345000 | 6292400000 | 1996900000 |  | IV | Yes |
| PF11_0034/  PF10_0381 | PF3D7_1102200/PF3D7_1039100 | eCiJp |  | 6422500000 | 960310000 |  | IV | Yes |

**Supplementary Table S5:** Details of the JDP AlphaFold models and Protein Data Bank (PDB) IDs used to render full-length JDP and J domain molecular graphics (for Figure 2)

| **Protein Name** | **Uniprot ID** | **AlphaFold/PDB ID** |
| --- | --- | --- |
| DnaJ | P08622 | AF-P08622-F1, 5NRO |
| CbpA | P36659 | AF-P36659-F1, 2KQX |
| DjlA | P31680 | AF-P31680-F1 |
| E.coli DljB | P77381 | AF-P77381-F1 |
| DNAJA1 | P31689 | AF-P31689-F1, 2M6Y |
| DNAJB1 | P25685 | AF-P25685-F1, 1HDJ |
| Sec63 | Q9UGP8 | AF-Q9UGP8-F1 |
| DNAJB13 | P59910 | AF-P59910-F1 |
| PfHsp40 | Q8IL88 | AF-Q8IL88-F1 |
| PFE0055c | Q8I489 | AF-Q8I489-F1 |
| Pfj3 | Q8IJ23 | AF-Q8IJ23-F1 |
| RESA | Q8I0U6 | AF-Q8I0U6-F1 |
